# Vascular effects of perivascular adipose tissue-derived chemerin in obesity-associated cardiovascular disease

**DOI:** 10.1186/s12933-025-02814-5

**Published:** 2025-06-13

**Authors:** Andy W. C. Man, Ning Xia, Huige Li

**Affiliations:** Department of Pharmacology, University Medical Center, Johannes Gutenberg University, 55131 Mainz, Germany

**Keywords:** Obesity, eNOS, Perivascular adipose tissue, CMKLR1, ChemR23, Vascular remodeling

## Abstract

Perivascular adipose tissue (PVAT) is a unique and metabolically active adipose tissue that is adjacent to most systemic blood vessels. Healthy PVAT exerts anticontractile and anti-inflammatory effects, contributing to vascular protection. However, during obesity, PVAT becomes proinflammatory and profibrotic, exacerbating vascular dysfunction. Chemerin, a multifunctional adipokine, has emerged as a key regulator of vascular tone, inflammation, and remodeling. Although liver-derived chemerin dominates the circulating chemerin pool, PVAT-derived chemerin plays a more localized and functionally important role in vascular pathophysiology because of its proximity to the vessel wall. This review highlights the role of PVAT-derived chemerin in vascular health, the mechanistic involvement of PVAT-derived chemerin in certain aspects of obesity-associated cardiovascular diseases, and the therapeutic potential of targeting PVAT-derived chemerin.

## Research Insights

**What is currently known about this topic?**

- PVAT regulates vascular function by secreting adipokines and vasoactive factors.
- PVAT dysfunction in obesity promotes inflammation and vascular remodeling.
- Chemerin is an adipokine linked to hypertension and cardiovascular diseases.

**What is the key research question?**

**How does PVAT-derived chemerin contribute to vascular dysfunction in obesity?**

**What is new?**

- PVAT-derived chemerin plays a greater role in hypertension than liver-derived chemerin.
- Chemerin promotes endothelial dysfunction, oxidative stress, and vascular remodeling.
- Targeting PVAT-derived chemerin may be a potential therapeutic strategy.

**How might this study influence clinical practice?**

- PVAT-derived chemerin could be a novel target for treating obesity-related hypertension.

## Introduction

Cardiovascular diseases remain the leading cause of mortality worldwide [1, 2], with a significant proportion of cases linked to obesity and metabolic disorders [3]. Obesity contributes to vascular dysfunction through systemic inflammation, oxidative stress, and dysregulated adipokine signaling [4, 5]. Among the various adipose tissues, perivascular adipose tissue (PVAT) is uniquely positioned adjacent to most blood vessels and plays an active endocrine role in vascular physiology and pathology [6, 7].

Under healthy conditions, PVAT exerts anticontractile and anti-inflammatory effects [7]. However, PVAT becomes proinflammatory and profibrotic, contributing to endothelial dysfunction and vascular remodeling during obesity [8–10]. Chemerin, a multifunctional adipokine, has recently garnered attention because of its dual roles in metabolic regulation and vascular homeostasis [11–13]. While liver-derived chemerin dominates the circulating pool, the expression of chemerin has recently been reported in the vasculature, both in the blood vessel wall and its surrounding PVAT [14, 15]. Emerging evidence suggests a critical role for PVAT-derived chemerin in obesity-induced hypertension and vascular remodeling [15]. This review highlights the emerging role of PVAT-derived chemerin for vascular health and summarizes recent findings on its involvement in certain aspects of obesity-related cardiovascular diseases.

## PVAT 

PVAT is the fat surrounding the blood vessel and is directly adjacent to the vessel wall. Although historically regarded as merely structural support, PVAT is now widely recognized as an endocrine organ.

### PVAT composition

PVAT is a highly heterogeneous adipose tissue that contains adipocytes, blood cells, capillaries, stem cells, immune cells, and nerves. PVATs exhibit regional genotypic, phenotypic, and functional differences in different anatomical locations in the vascular system [16, 17]. By having different proportions of white, brown, or beige adipocytes, PVAT can be white adipose tissue (WAT)-like, brown adipose tissue (BAT)-like, or mixed. In rodents, the PVAT of the thoracic aorta is BAT-like, whereas WAT-like PVAT surrounds smaller arteries, such as the mesenteric, carotid, and femoral arteries. The abdominal aorta is surrounded by a beige PVAT [16, 17].

White adipocytes in the PVAT mainly perform lipid storage and endocrine functions [18]. Brown adipocytes contain multilocular lipid droplets and a high density of mitochondria, are metabolically active and are responsible for thermogenesis [19]. Brown adipocytes have the highest expression and white adipocytes have the lowest expression of uncoupling protein 1 (UCP1) [20]. UCP1 expression is negatively associated with reactive oxygen species (ROS) production in adipocytes, implying that the hierarchy of ROS production in adipocytes is white > beige > brown [21]. White adipose tissue has a significant proinflammatory profile, defined by increased immune cell infiltration and adipokine synthesis linked to inflammatory regulation [22]. In contrast, BAT is generally more resistant to obesity-induced local inflammation [23]. Nevertheless, severe obesogenic stimuli can promote a proinflammatory milieu within BAT [23, 24]. During obesity, metabolically overloaded brown adipocytes release damaged lipids and mitochondrial components, driving the accumulation of lipid-scavenging macrophages in BAT. These immune cells actively reprogram brown adipocytes, facilitating the whitening of BAT toward a WAT-like state [25].

Recent findings suggest that adipocytes in the anterior thoracic aortic PVAT predominantly originate from progenitor cells expressing smooth muscle protein 22-alpha (SM22α) and that adipocytes in the lateral regions of thoracic PVAT exhibit markers of both SM22α^+^ and myogenic factor 5 (Myf5^+^) lineages [26–28]. In contrast, another study reported that fibroblastic progenitor cells, rather than vascular smooth muscle cells (VSMCs, contribute to adipogenesis in thoracic PVAT [29]. The developmental origins of adipocytes in abdominal periaortic PVAT remain less well characterized. However, evidence suggests that these adipocytes may share a common origin with SM22α^+^ and peroxisome proliferator-activated receptor gamma-positive (PPARγ^+^) VSMCs, as SM22α-Cre-mediated PPARγ deletion completely abolishes the formation of abdominal periaortic PVAT [30]. Moreover, the same genetic deletion also leads to a marked reduction in mesenteric PVAT but not in other white adipose depots, further suggesting a shared developmental lineage between mesenteric PVAT and SM22α^+^/PPARγ^+^ VSMCs [30]. In contrast, other reports have indicated that mesenteric PVAT shares developmental origins that are more closely aligned with visceral WAT [31, 32].

### PVAT secretion and PVAT dysfunction

As in all other adipose tissues, PVAT produces and secretes growth factors, hormones, adipokines, microRNAs (miRNAs), and extracellular microvesicles to modulate multiple biological processes [33, 34]. Moreover, paracrine crosstalk between PVAT and its connecting vessels plays a crucial role in regulating vascular inflammation and remodeling [31]. PVAT-derived proinflammatory and/or anti-inflammatory vasoactive substances modulate vascular tone, vascular inflammation, oxidative stress, and VSMC proliferation and migration [17, 35]. Owing to their anticontractile or contractile functions, these PVAT-derived substances are also called PVAT-derived relaxing factors (PVRFs) and PVAT-derived contracting factors (PVCFs), respectively [34]. PVRFs include adiponectin [36], apelin [37], angiopoietins [38], vaspin [39], hydrogen peroxide (H_2_O_2_) [40], hydrogen sulfide (H_2_S) [41], prostaglandins [42, 43], nitric oxide (NO) [44], and angiotensin [1–7, 45], whereas PVCFs include calpastatin [46], chemerin [47], serotonin [48], norepinephrine [49], angiotensin II, and reactive oxygen species (ROS) [50]. Under normal physiological conditions, PVAT releases PVRFs, adipokines, and cytokines, which are mainly anti-inflammatory and promote vascular function and homeostasis. PVAT-derived factors may modulate vascular function through two distinct mechanisms: endothelium-independent and endothelium-dependent pathways [44, 51]; they may diffuse directly into the endothelium or through the vasa vasorum or tiny media conduit networks that link the medial layer to the underlying adventitia [16, 52, 53].

Recent data indicate that the vascular wall and PVAT interact reciprocally, with the vascular wall influencing PVAT through paracrine signals that alter the secretory phenotype of PVAT [54]. In obesity, oxidative products released from the blood vessel wall may diffuse to PVAT and lead to the upregulation of the expression of adiponectin, which in turn exerts antioxidant effects on the adjacent vessel wall [55]. Therefore, PVAT can act as a protective mechanism against oxidative stress in blood vessels. On the other hand, proinflammatory cytokines secreted from the vessel wall, such as interleukin-6 (IL-6), tumor necrosis factor-alpha (TNF-α), interferon-γ (IFN-γ), inhibit the differentiation of preadipocytes into mature adipocytes and lead to a reduction in lipid droplet accumulation in PVAT [56]. Typically, adipocytes are uniform in size and content in the same region of PVAT [57]. However, in inflamed blood vessel walls, adipocytes with a gradient in size form in PVAT, with tiny, undifferentiated preadipocytes with lower lipid content proximal to the vessel surrounded by larger, fat-filled white adipocytes distal to the vessel wall [56]. In our previous study, acetylcholine-induced NO-mediated vasodilation was preserved in PVAT-free aortas but impaired in the PVAT-intact aortas of obese mice [58]. In addition, the transplantation of PVAT from high-cholesterol diet-fed apolipoprotein E knockout (ApoE^−/−^) mice to normal control diet-fed ApoE^−/−^ mice resulted in a significant increase in atherosclerosis development [59]. These findings suggest that PVAT significantly contributes to the progression of vascular dysfunction and metabolic disease complications.

During obesity, PVAT dysfunction leads to an imbalance in PVAT-derived factor secretion, resulting in the impairment of vascular function and homeostasis [60–63]. The loss of PVAT anticontractile function has been reported in different animal models of metabolic and cardiovascular diseases, including hypertension, obesity, and diabetes [8, 64, 65]. In addition to phenotypic changes, PVAT switches to a proinflammatory profile, and PVAT adipocytes generate PVCFs and adipokines such as leptin [36], visfatin/nicotinamide phosphoribosyltransferase (NAMPT) [66], resistin [67], lipocalin 2 [68], and chemerin [15], which diffuse to the adjacent blood vessel wall and trigger vasoconstriction, endothelial dysfunction, and vascular remodeling. In addition, proinflammatory cytokines, including IL-6, TNF-α, IFN-γ, and monocyte chemoattractant protein-1 (MCP-1), are secreted by immune cells in PVAT and contribute to local inflammation [69, 70]. Recent studies have revealed that PVAT can also secrete different types of extracellular vesicles, including exosomes and microvesicles [71, 72]. These extracellular vesicles play important roles in regulating vascular functions by transporting enclosed messengers, including proteins, lipids, noncoding RNAs, and miRNAs, for intercellular cross-talk [73]. Circulating exosomal miRNAs secreted in the form of adipokines can regulate gene expression locally or distantly [74]. These extracellular vesicles are crucial for crosstalk between PVAT and cells in the vasculature, including endothelial cells, VSMCs, and macrophages [72, 73, 75]. A recent study revealed that encapsulated miRNAs secreted from PVAT, such as miR-221-3p, can trigger early vascular remodeling in vascular inflammation [71]. Additionally, PVAT-derived exosomes reduce macrophage foam cell formation through the miR-382-5p-mediated upregulation of cholesterol efflux transporter expression (76).

## Chemerin and its receptors

### Chemerin expression

Chemerin was first discovered in psoriatic lesions, where its gene expression increased after topical exposure to the retinoid tazarotene; hence, it was first named tazarotene-induced gene 2, which was later renamed retinoic acid receptor responder 2 (*RARRES2*) [77]. Chemerin synthesis begins with preprochemerin [78]; it is a small protein comprising 163 amino acids encoded by the *RARRES2* gene [79]. Preprochemerin undergoes proteolytic cleavage to remove its 20-amino-acid signal peptide and is secreted as a biologically inactive isoform, prochemerin, into the bloodstream [80].

Chemerin isoforms are generated from prochemerin via proteolytic C-terminal cleavage by proteases such as plasmin, carboxypeptidases, and serine proteases, generating isoforms with distinct biological activities [81–85]. Isoform diversity depends on cleavage sites, tissue origin, and detection methods [86]. While some isoforms are highly active, shorter variants are often inactive or antagonistic [87]. Inactive isoforms include chemerin-155, produced via elastase cleavage, and chemerin-154, which lacks chemoattractant activity and fails to induce intracellular calcium mobilization—indicating functional inactivity [88, 89]. In contrast, chemerin-156 and chemerin-157 are active forms, with chemerin-157 showing the highest potency in stimulating chemotaxis and receptor signaling [90]. Proteolytic sites are conserved between human and murine chemerin, supporting translational studies [88]. Isoform quantification in serum and tissues is achievable using liquid chromatography–mass spectrometry (LC–MS) techniques and isoform-specific ELISAs validated for both species [89]. Total chemerin levels and active/inactive isoform ratios vary across adipose depots; however, the isoform composition in specific PVAT regions remains poorly defined. Mapping protease expression profiles within PVAT will be essential to understand local chemerin processing and its role in vascular and metabolic regulation.

Chemerin is highly expressed in the liver [91]. The hepatic knockdown of chemerin in rats results in an almost complete absence of circulating chemerin, suggesting that the liver is the predominant source of circulating chemerin [92, 93]. However, chemerin is also highly expressed in both brown and white adipocytes in adipose tissue [91, 94]. Two studies, including one from our group, have recently shown chemerin expression in the vasculature, both in the blood vessel wall and its surrounding PVAT [14, 15].

To date, the regulatory mechanisms underlying chemerin expression in different cell types remain obscure. In a recent study, the proximal fragment (− 252 to + 258 bp) of the *RARRES2* gene promoter was identified as a key regulator of transcription [95]. Acute-phase cytokines (IL-1β and oncostatin M) specifically induce chemerin expression in mouse adipocytes but have little effect on hepatocytes [95]. These results suggest that chemerin expression is regulated in a cell type-specific manner.

### Liver-derived versus PVAT-derived chemerin

The important role of chemerin in blood pressure regulation can be seen in experiments with antisense oligonucleotides (ASOs) (Table 1). The administration of whole-body chemerin ASOs results in the near-complete depletion of circulating chemerin levels, an effect that is associated with a reduction in mean arterial pressure (MAP) of approximately 7 mmHg in normotensive Sprague‒Dawley (SD) rats [92]. In high-fat diet (HFD)-fed Dahl salt-sensitive (DSS) rats with obesity and hypertension, the effect is markedly stronger, resulting in a MAP reduction as high as 29 mmHg [93]. The administration of an *N*-acetylgalactosamine (GalNAc)-ASO, which destroys chemerin mRNA specifically in the liver, reduces plasma chemerin by 90% and 96% in lean SD and obese DSS rats, respectively [92, 93]. However, liver-specific GalNAc-ASOs do not reduce blood pressure in SD rats [92]. In hypertensive HFD-fed DSS rats, GalNAc-ASO lowers MAP by only 6 mmHg [93]. These results suggest that blood pressure is not directly correlated with plasma chemerin levels and that chemerin from remote sources is not the major player in local vascular effects. Instead, extrahepatic sources, such as PVAT or the vascular wall itself, may play a more dominant role in blood pressure regulation. Indeed, chemerin mRNA and protein are found in both the smooth muscle layer of the rat aorta and in PVAT [14]. Moreover, PVAT has been shown to be the main source of vascular chemerin [13]. Chemerin expression is significantly greater in PVAT than in the vascular wall, whereas the expression of the chemerin receptor CMKLR1 is markedly greater in the tunica media than in PVAT [13]. PVAT-derived chemerin may have direct effects on VSMCs owing to the close proximity and absence of a mechanical barrier between PVAT and the blood vessel wall [96]. Consistent with this concept, the results of functional studies demonstrated that the CMKLR1 antagonist CCX832 inhibits norepinephrine- and serotonin-induced vasoconstriction in PVAT-intact rat aortae but not in PVAT-free aortae [13]. Thus, the majority of functionally relevant endogenous chemerin in the vasculature is derived from PVAT rather than the vascular wall, at least under this experimental setting. Notably, the blood pressure-reducing effects of whole-body ASO are associated with a 94% reduction in PVAT chemerin [92]. Collectively, the available evidence suggests that PVAT-derived chemerin plays a crucial role in vascular function and blood pressure regulation.

**Table 1:** The role of chemerin in blood pressure regulation

Animals	Diet	Chemerin ASO	Circulating chemerin	PVAT chemerin	MAP reduction	References
SD	NCD	whole body ASO	Undetectable	↓94%	7 mmHg	[92]
SD	NCD	Liver ASO	↓ 90%	↔	↔	[92]
DSSR	NCD	whole body ASO	Undetectable	n.d	10 mmHg	[14]
DSSR	HFD	whole body ASO	Undetectable	n.d	29 mmHg	[93]
DSSR	HFD	Liver ASO	↓ 96%	n.d	6 mmHg	[93]

ASO, antisense oligonucleotides; DSSR, Dahl salt-sensitive rats; GalNAc, N-acetylgalactosamine; HFD, high-fat diet; MAP, mean arterial pressure; NCD, normal control diet; n.d., no data; SD, Sprague‒Dawley rats; ↔, no significant changes

### Chemerin receptors

**Fig. 1:**
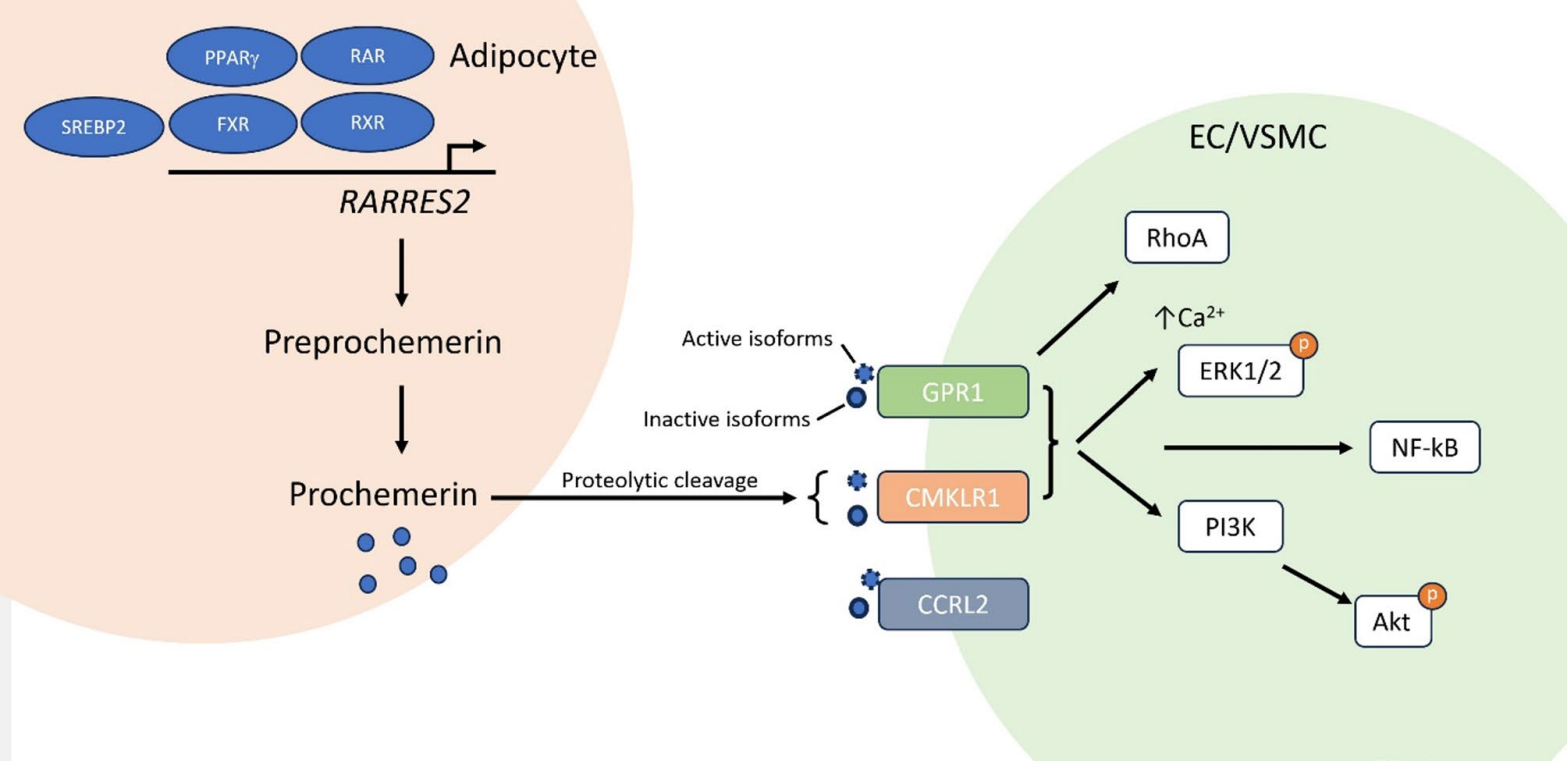
Chemerin expression, receptor activation, and signaling cascades. Preprochemerin is encoded by the *RARRES2* gene. Chemerin expression is regulated by various modulators. Preprochemerin undergoes proteolytic cleavage to remove its 20-amino-acid signal peptide and is secreted as a biologically inactive isoform, prochemerin, from adipocytes into the bloodstream. There are different serine proteases in different tissues that cleave prochemerin into different active or inactive chemerin isoforms that bind to chemerin receptors present on the cell membrane of different target cells in the vessel wall. The binding of chemerin to GPR1 or CMKLR1 triggers calcium mobilization, ERK1/2 and PI3K/Akt pathway activation, and NF-κB activation. CCRL2 acts as a chaperone protein that concentrates and presents chemerin to CMKLR1 and GPR1. Akt, protein kinase B; CCRL2, CC motif chemokine receptor-like 2; CMKLR1, chemokine-like receptor 1; EC, endothelial cell; ERK1/2, extracellular signal-regulated kinase 1/2; FXR, farnesoid X receptor; GPR1, G protein-coupled receptor 1; NF-κB, nuclear factor kappa B; PI3K, phosphoinositide 3-kinase; PPARγ, peroxisome proliferator-activated receptor gamma; RAR, retinoic acid receptor; *RARRES2*, retinoic acid receptor responder 2; RXR, retinoid X receptor; SREBP2, sterol regulatory element-binding protein 2; VSMC, vascular smooth muscle cell

Currently, there are three known chemerin receptors (Fig. 1). Chemerin primarily acts through the G protein-coupled orphan receptor chemokine-like receptor 1 (ChemR23, CMKLR1) [78, 79]. CMKLR1 is the most widely studied chemerin receptor. *CMKLR1* is expressed in adipose tissues, dendritic cells, endothelial cells, macrophages, monocytes, heart, lungs, muscle, placenta, skin, and spleen [11]. Chemerin binds to CMKLR1 and leads to the activation of G_i_ and decreases cyclic adenosine monophosphate (cAMP) levels and increases intracellular calcium release, which results in the phosphorylation of the extracellular signal-regulated kinase 1/2 (ERK1/2) and phosphoinositide-3-kinase (PI3K)/protein kinase B (Akt) pathways and the activation of nuclear factor kappa B (NF-κB) [97].

Another receptor of chemerin is G protein-coupled receptor 1 (GPR1), also known as chemokine-like receptor 2 (CMKLR2) [98]. GPR1 has approximately 40% sequence homology with CMKLR1 [99]. GPR1 is expressed in the placenta, ovaries, testicles, skin, adipose tissue, skeletal muscle, and brain [100, 101]. GPR1 has more ligands than just chemerin; however, it binds chemerin with high affinity, resulting in relatively weak G_i_-dependent signaling [102]. However, the role of GPR1 as a chemerin receptor remains unclear. Chemerin may bind to GPR1, resulting in modest calcium release [98] and G_i/o_-dependent RhoA signaling [103]. It has also been shown that chemerin elicits potent constrictor actions via CMKLR1 but not GPR1 [104]. On the other hand, a recent study revealed that mice lacking GPR1 exhibited reduced glucose-stimulated insulin secretion and increased glucose levels during a pyruvate tolerance test [105], suggesting a role for GPR1 in regulating glucose homeostasis during obesity.

The third chemerin-binding receptor is CC motif chemokine receptor-like 2 (CCRL2), which is believed to be a chaperone protein that concentrates and presents chemerin to CMKLR1 and GPR1 [106]. CCRL2 does not internalize chemerin or transduce downstream signals [80]. CCRL2 is expressed in adipose tissues, breasts, dendritic cells, lungs, macrophages, microglia, neutrophils, and placenta [80]. GPR1 and CCRL2 appear to be involved in the peculiar manifestations of chemerin, but most chemerin effects seem to be dependent on CMKLR1.

## Vascular effects of chemerin

### Effects on the endothelium

The proliferation and migration of endothelial cells lead to angiogenesis, whereas inflammation and hypoxia may initiate hypoxia-inducible factor (HIF)-driven angiogenesis in vascular complications such as atherosclerosis [107]. During obesity, increased levels of inflammatory cytokines, such as IL-6 and TNF-α, augment *CMKLR1* expression and increase monocyte attachment to endothelial cells [108–110]. Chemerin/CMKLR1 activates ERK1/2 and p38 mitogen-activated protein kinase (MAPK) pathways in human umbilical vein endothelial cells and stimulates angiogenesis [108].

In addition, chemerin is involved in the activation of matrix metalloproteinases-2/9 (MMP-2/9) in a dose-dependent manner, thus modulating the degradation of the extracellular matrix during endothelial cell proliferation and migration [108]. Moreover, the chemerin/CMKLR1 axis promotes angiogenesis by inducing oxidative stress-mediated autophagy via AMP-activated protein kinase α (AMPKα) [111]. Treatment with the mitochondria-targeted antioxidant Mito-TEMPO or *CMKLR1* knockdown reduced chemerin-induced ROS generation and ameliorated the upregulation of autophagy-related gene expression [111]. Interestingly, knocking down CMKLR1, but not CCRL2, completely inhibited the chemerin-induced migration and proliferation of endothelial cells, thus reversing angiogenesis in vitro [112].

Chemerin attenuated endothelial nitric oxide synthase (eNOS) activity and diminished NO production in cultured human microvascular endothelial cells [109]. In rat aortic rings incubated with chemerin, vascular NO/cyclic guanosine monophosphate (cGMP) signaling and vascular relaxation were significantly reduced. Chemerin leads to eNOS uncoupling, increased superoxide production, and reduced NO production, which are associated with decreased soluble guanylyl cyclase (sGC) activation and cGMP production [113]. These results suggest that increased chemerin expression is involved in obesity-related endothelial dysfunction and angiogenesis.

### Effects on VSMCs

The synthetic CMKLR1 agonist chemerin-9 has been shown to induce concentration-dependent vasoconstriction in several vascular beds, including isolated rat aorta, rat intrapulmonary arteries, human saphenous vein, and human resistance arteries [47, 104, 114–116]. This vasoconstrictive effect is mediated by CMKLR1 activation and is calcium-dependent, indicating the involvement of intracellular calcium mobilization in the contractile response [115]. In addition, endogenous chemerin derived from PVAT has been shown to enhance vasoconstrictor responses to norepinephrine and serotonin, further supporting its role in modulating vascular tone [13]. These vasoconstrictive actions contribute to the blood pressure–elevating effects of chemerin, underscoring its relevance in the pathophysiology of vascular dysfunction and hypertension.

VSMC proliferation and migration are critically involved in the pathophysiology of vascular remodeling [117]. In human atherosclerotic lesions, positive chemerin staining has been observed in VSMCs, PVAT, and foam cells, whereas chemerin expression has been shown to be positively correlated with the severity of atherosclerosis [118]. In a metabolic hypertension rat model, the expression of chemerin and its receptor CMKLR1 are upregulated in the thoracic aorta and mesenteric arteries, accompanied by enhanced vascular remodeling in vivo [119]. In HFD-fed ApoE^−/−^ mice, aortic plaque formation and vascular remodeling are positively correlated with the expression of chemerin and its downstream targets, including MAPK [120]. The adenovirus-mediated knockdown of chemerin normalizes the expression of inflammatory cytokines and significantly ameliorates aortic atherosclerosis and vascular remodeling, suggesting that chemerin may promote atherosclerosis progression via the MAPK pathway [120]. Chemerin has been shown to increase ROS production and stimulate arterial smooth muscle cell proliferation and migration in vitro [90]. The proinflammatory and proliferative effects of chemerin in VSMCs are likely mediated by the activation of NADPH oxidase and redox-sensitive MAPK signaling, as these effects are prevented by the inhibition of NADPH oxidase [109]. In addition, chemerin has been shown to increase pulmonary artery smooth muscle cell proliferation in an endothelin-1-dependent manner, whereas endothelin-1 exacerbates chemerin-induced thoracic aorta and pulmonary artery smooth muscle cell migration in vitro [121].

Moreover, clinical studies have reported a significant positive correlation between circulating chemerin levels and arterial stiffness in obese patients [122, 123]. Consistent with these clinical observations, the enhanced vascular remodeling in adipocyte-specific eNOS-knockout mice has been shown to be associated with increased chemerin expression in PVAT as well as elevated circulating chemerin levels [15]. Moreover, incubation of mouse aorta with chemerin-containing serum has been shown to increase the expression of vascular remodeling-related genes, an effect that is prevented by a chemerin-neutralizing antibody [15]. These results collectively suggest that chemerin is involved in the pathophysiology of vascular remodeling.

### Effects on PVAT function

Research on the impact of chemerin on PVAT function in obesity remains limited to date. The systemic knockout of CMKLR1 significantly reduces the number of macrophages in adipose tissues, including PVAT, and has an adverse effect on the phenotypic switching of adipose tissue macrophages in the PVAT of hyperlipidemic mice during atherosclerosis [124]. In cultured 3T3-L1 adipocytes, chemerin has been shown to play an important role in regulating adipogenesis and adipocyte metabolism [94]; however, this role has not yet been investigated in PVAT. In a recent study, knockdown of chemerin mRNA has been shown to reduce differentiation and fatty acid uptake in brown adipocytes [125]. Moreover, despite increased BAT weight, mitochondrial content and function are significantly lower in the BAT of chemerin global knockout mice than in that of control mice [125]. In addition, compared with control mice, chemerin global knockout mice exhibit reduced body temperature, oxygen consumption, carbon dioxide production, energy expenditure, and respiratory exchange ratio. These results suggest that chemerin plays a pivotal role in BAT differentiation and thermogenesis. As mentioned above, chemerin is known to reduce NO production in endothelial cells [109, 113]. Therefore, chemerin may also affect the expression and function of eNOS in PVAT adipocytes. On the other hand, our recent study revealed that the adipocyte-specific knockout of eNOS enhances chemerin expression, indicating that NO may be a crucial factor limiting chemerin expression in PVAT [15]. Indeed, there are two nuclear receptors that heterodimerize with retinoid X receptor (RXR) and one nuclear regulatory factor that affects chemerin expression [126, 127]. Moreover, the promoter of the chemerin gene includes both a PPARγ-binding sequence and a sterol regulatory element-binding protein 2 (SREBP2)-binding site [95]. NO-triggered signaling pathways may regulate chemerin expression through these elements.

## Proinflammatory versus anti-inflammatory effects

Chemerin has been implicated in both pro- and anti-inflammatory processes, with several studies supporting its pro-inflammatory potential. In addition to its role in activating NF-κB signaling, chemerin facilitates the recruitment of leukocytes to sites of inflammation. In subcutaneous adipose tissue-specific chemerin-knockdown mice, the expression of tissue inhibitor of metalloproteinases-1 (TIMP1) is reduced, resulting in increased adipogenesis and improved glucose metabolism, concomitant with decreased macrophage infiltration [128]. These findings suggest that chemerin may regulate adipose tissue remodeling through its effects on TIMP1 expression and immune cell recruitment.

Chemerin is known to induce calcium mobilization and chemotaxis in macrophages and immature dendritic cells via the CMKLR1 receptor, which is expressed on various immune cells, including immature dendritic cells, macrophages, leukocytes, and natural killer (NK) cells [78, 129]. During obesity, chemerin appears to exacerbate local and systemic inflammation, a key contributor to insulin resistance and adipose tissue expansion; it has been shown to recruit circulating dendritic cells to visceral adipose tissue. The activation of Toll-like receptor 9 (TLR9) in dendritic cells leads to the secretion of type I interferons, thereby initiating a proinflammatory response in macrophages [130]. Adipose tissue macrophages promote the recruitment, proliferation, and differentiation of adipocyte progenitor cells by secreting osteopontin [131]. Preadipocytes cultured in conditioned medium from activated macrophages exhibit increased extracellular matrix remodeling [132]. In mice fed a HFD, macrophages accumulate around necrotic adipocytes, forming “crown-like structures” [133]. Collectively, these observations suggest that chemerin promotes obesity-induced adipose tissue inflammation by enhancing immune cell infiltration into PVAT and other adipose tissues.

In contrast, several studies have documented the anti-inflammatory roles of chemerin and its receptor CMKLR1. Chemerin has been reported to suppress the production of proinflammatory cytokines, thereby exerting an immunomodulatory effect [134]. In a murine peritonitis model, chemerin inhibited monocyte and neutrophil recruitment and reduced the expression of proinflammatory mediators [135]. Furthermore, CMKLR1 activation in macrophages has been shown to induce the expression of interleukin-10 (IL-10), an anti-inflammatory cytokine [136]. In a mouse model of acute lung inflammation induced by lipopolysaccharides (LPS), chemerin was shown to exert potent anti-inflammatory effects by reducing both cytokine production and neutrophil infiltration via a CMKLR1-dependent mechanism [137]. Similarly, chemerin attenuates inflammation by suppressing the production of CC-chemokine ligand 2 (CCL2) in a murine model of allergic asthma [138]. In addition, chemerin also modulates NK cell activity, which plays a pivotal role in the early innate immune response and resolution of inflammation [129]. Chemerin has been shown to facilitate the recruitment of dendritic cells and NK cells to inflamed tissues, where they modulate adaptive immune responses [139].

The contradictory effects of chemerin may be caused by the differential activity of its isoforms, which arises through proteolytic processing of the chemerin precursor. The anti-inflammatory potential of chemerin appears to depend on its cleavage into specific isoforms, a process determined by the activity of serine and cysteine proteases in the tissue microenvironment [140]. Certain chemerin isoforms preferentially induce anti-inflammatory macrophage phenotypes [137], whereas others promote proinflammatory polarization [141]. In addition, proteases present in mast cells and neutrophils can generate chemerin isoforms that are inactive, nonchemotactic, or even anti-inflammatory [111, 142]. A notable example is chemerin15 (C15), a chemerin-derived peptide with well-characterized anti-inflammatory properties. C15 has been shown to inhibit neutrophil recruitment by suppressing integrin activation and to enhance the clearance of neutrophils and apoptotic cells, thereby promoting the resolution of inflammation [143].

## Chemerin in obesity

### Changes in chemerin in humans during obesity

Dysregulation of chemerin is consistently observed in human obesity. Clinical studies report that serum chemerin levels correlate positively with body mass index (BMI), triglycerides, and blood pressure in healthy adults [144]. Obese individuals display significantly elevated circulating chemerin levels compared to lean controls [145, 146]. In addition, concentrations of the bioactive isoform chemerin-157 are markedly elevated—by up to 1000-fold—in omental and subcutaneous adipose tissues of obese individuals, and are positively associated with C-reactive protein (CRP), a marker of systemic inflammation [89].

### Changes in chemerin in animal models during obesity

Similar trends are evident in animal models of obesity. Circulating chemerin levels increase in proportion to visceral fat accumulation, aortic stiffness, and blood pressure in rodents [14, 92, 147]. Obese mouse models, such as db/db mice and mice on HFD, exhibit twofold higher serum chemerin levels than lean controls [147, 148]. Conversely, fasting or caloric restriction reduces chemerin levels in rodents [149, 150]. Obesity also alters chemerin receptor signaling; chemerin and CMKLR1 expression in PVAT are approximately twofold higher in HFD-fed rats than in controls [151].

However, findings on tissue-specific expression are inconsistent. In db/db mice, one study reported reduced chemerin expression in visceral WAT [152], while another found increased chemerin expression in both subcutaneous and visceral WAT in diabetic *Psammomys obesus* compared to normoglycemic controls [91]. Moreover, chemerin-knockout mice show complex phenotypes: chemerin deletion exacerbates HFD-induced obesity and adiposity while improving blood lipid profiles [153]. In aged male mice, chemerin deletion also abolishes exercise-induced benefits on weight reduction, WAT browning, and lipid metabolism [153], indicating a context-dependent role.

## Sex differences in chemerin expression and function

### Sex differences in chemerin in humans

Sex-specific differences in chemerin expression have been observed in humans, though findings are inconsistent. Some studies report higher chemerin mRNA expression [154, 155] and serum concentrations [156] in healthy women than in men. Additionally, nocturnal serum chemerin levels are elevated in obese women but not in obese men, relative to lean counterparts [157]. Contrarily, other reports have found no significant sex differences in plasma chemerin levels [158], and data from a Japanese cohort with metabolic syndrome or type 2 diabetes indicated higher chemerin levels in males than in females [159].

### Sex differences in chemerin in animal models

In wild-type rats, plasma and tissue chemerin concentrations are lower in females than in males [160]. Sex also modifies the physiological response to chemerin deletion. Following treatment with DOCA-salt and uninephrectomy, male chemerin-KO rats exhibit a greater elevation in blood pressure than wild-type males, whereas female KO rats show reduced blood pressure compared to wild-type females under the same conditions [160]. These findings suggest a sex-dependent role of chemerin in cardiovascular regulation in experimental hypertension.

### Potential mechanisms of sex differences in chemerin expression

It remains unclear whether the observed sex differences in chemerin expression are directly driven by sex hormones. In a small clinical trial involving 32 individuals, an inverse correlation between estradiol (E_2_) and serum chemerin levels was observed [158]. However, this association does not necessarily imply a causal effect of estradiol in the suppression of chemerin expression. An alternative interpretation is that chemerin may negatively regulate estradiol production, as demonstrated in cultured bovine ovarian granulosa cells [161]. In the porcine endometrium, the influence of estradiol on chemerin secretion appears to be context-dependent, with increased chemerin secretion observed during the period of the maternal recognition of pregnancy and reduced levels during embryo implantation [162]. The role of estradiol in regulating chemerin expression in the liver—the principal source of circulating chemerin—has not been elucidated. Furthermore, in ex vivo studies using human subcutaneous adipose tissue explants, no direct effect of 17β-estradiol on chemerin expression has been observed [163]. The interaction between testosterone and chemerin is also poorly characterized. In the same adipose tissue explant model, testosterone treatment does not alter chemerin expression [163]. However, the genetic ablation of chemerin in mice leads to increased serum testosterone concentrations under HFD conditions but not under NCD conditions, indicating a potential inhibitory effect of chemerin on androgen synthesis in a diet-dependent manner [164].

Gonadectomy studies provide additional insight. Ovariectomy in adult female rats decreases whereas orchidectomy in adult male rats increases chemerin expression in WAT [165]. Nonetheless, whether these effects are directly mediated by sex steroids or involve other hormonal or metabolic alterations remains to be determined. Notably, the pubertal increase in gonadal steroid levels is associated with a marked reduction in WAT chemerin expression in both male and female SD rats, suggesting the developmental regulation of chemerin expression by sex hormones [165].

In summary, current evidence supports a complex and context-dependent relationship between sex steroids and chemerin, with differential effects observed across tissues, developmental stages, and metabolic conditions. The mechanistic basis for the sex-specific regulation of chemerin remains to be fully elucidated and warrants further investigation.

## Potential research directions and therapeutics targeting PVAT-derived chemerin in obesity-associated cardiovascular disease

Although chemerin is implicated in the pathogenesis of obesity-related metabolic and cardiovascular disorders, no pharmacological agents that specifically target the chemerin/CMKLR1 axis, including PVAT-derived chemerin, are currently available. However, a variety of strategies have been employed in experimental models to modulate chemerin signaling, which may offer promising therapeutic avenues for the management of chemerin-associated complications in humans.

### Potential models for studying PVAT-derived chemerin

Rodent models with global deletion of chemerin or its receptor CMKLR1 are available [124, 160, 166, 167]. However, to elucidate the specific functions of PVAT-derived chemerin, there is a critical need to develop PVAT-specific knockout models targeting either chemerin or CMKLR1. Currently, the developmental origin and differentiation pathways of PVAT adipocytes remain incompletely understood. Emerging evidence suggests that PVAT, as a distinct anatomical depot, may contain adipocytes derived from unique progenitor cell populations, contributing to the morphological and functional characteristics that distinguish PVAT from other adipose tissues [168–170]. As discussed above, PVAT adipocytes may originate from SM22α^+^ and/or Myf5^+^ progenitor cells, supporting the feasibility of generating PVAT-specific gene knockout models to enable more precise functional studies—although such an approach remains challenging. Progress in this area depends on the identification of lineage-specific markers and progenitor populations unique to PVAT, which are urgently needed to facilitate the development of PVAT-selective genetic tools.

### Chemerin antisense oligonucleotides

ASOs are short, single-stranded oligodeoxynucleotides that inhibit protein translation through sequence-specific hybridization to target mRNA, leading to RNA degradation via RNase H-mediated mechanisms. In a rat model, the systemic administration of a whole-body chemerin-targeting ASO resulted in a nearly complete reduction in chemerin protein expression in PVAT, other adipose tissues, the liver, and plasma and was associated with a significant decrease in blood pressure [92]. In contrast, a GalNAc-conjugated chemerin ASO designed for hepatocyte-specific delivery selectively reduces hepatic chemerin expression but fails to lower systemic blood pressure, suggesting that extrahepatic sources may be more relevant for chemerin-mediated vascular effects [92, 93]. Efforts to achieve the adipocyte-specific knockdown of chemerin using nanoparticles conjugated with an adipose-homing peptide have shown limited success. Although the nanoparticles successfully encapsulate and deliver ASOs, dominant hepatic uptake impedes adipose-specific targeting, thus limiting the efficacy of this approach [14, 171]. Overall, these findings highlight the technical challenges associated with developing PVAT-specific chemerin ASO delivery systems and underscore the need for improved targeting strategies to dissect the tissue-specific functions of chemerin in cardiometabolic regulation.

### Chemerin analogs

Synthetic chemerin analogs may represent another potential therapeutic strategy for modulating chemerin receptor signaling. Chemerin-9, a stable nonapeptide corresponding to the C-terminus of chemerin-157, is an agonist of CMKLR1 [172]. Unlike full-length chemerin, which has both pro- and anti-inflammatory properties depending on context, chemerin-9 predominantly exerts anti-inflammatory and vasoprotective effects. In a recent study, chemerin-9 infusion was shown to significantly attenuate the development of abdominal aortic aneurysms in obese ApoE^−/−^ mice. This effect was associated with reduced inflammatory cell infiltration, neovascularization, and MMP expression within the aortic wall [173]. In another study, the systemic administration of chemerin-9 was shown to decrease aortic atherosclerotic lesion areas, an effect accompanied by a reduction in intraplaque macrophage content in vivo. Additionally, chemerin-9 suppresses TNF-α-induced monocyte adhesion to endothelial cells and attenuates the inflammatory phenotype of macrophages in vitro [174].

### CMKLR1 receptor antagonists

CCX832, developed by ChemoCentryx, is a selective antagonist of CMKLR1, with minimal affinity for the other two known chemerin receptors [47]. Multiple in vitro and in vivo studies have demonstrated that CCX832 effectively mitigates chemerin/CMKLR1 axis-mediated metabolic and cardiovascular dysfunction [47, 104, 175].

**Fig. 2:**
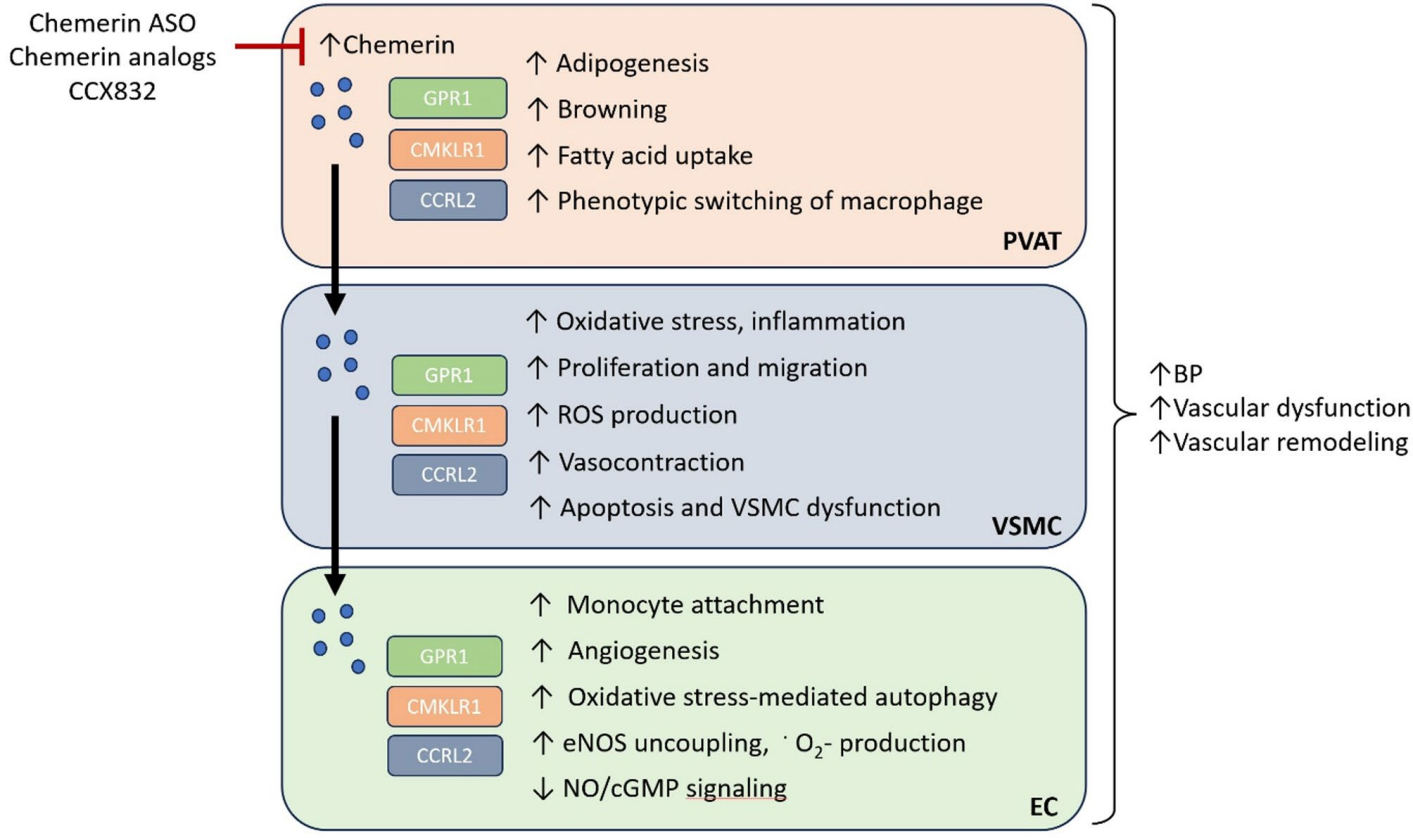
Vascular effects of PVAT-derived chemerin. During obesity, chemerin expression in perivascular adipose tissue (PVAT) is significantly increased. PVAT-derived chemerin affects all the cellular layers of the vascular wall, including endothelial cells (ECs), vascular smooth muscle cells (VSMCs) and PVAT itself, via chemerin receptors. These adverse effects lead to an overall increase in blood pressure (BP) and promote vascular dysfunction and remodeling. Potential treatment strategies include the use of chemerin antisense oligonucleotides (ASOs), inactive chemerin isoforms as competitors, and chemerin receptor inhibitors/antagonists, such as CCX832. CCRL2, CC motif chemokine receptor-like 2; cGMP, cyclic guanosine monophosphate; CMKLR1, chemokine-like receptor 1; GPR1, G protein-coupled receptor 1; eNOS, endothelial nitric oxide synthase; NO, nitric oxide; ^·^O_2_^−^, superoxide; ROS, reactive oxygen species

The pharmacological blockade of CMKLR1 using CCX832 has been shown to reduce body weight, plasma insulin and glucose levels, and vascular oxidative stress in obese mice; additionally, it significantly ameliorates chemerin-induced vascular inflammation [108, 175]. In addition to its systemic effects, CCX832 has been reported to inhibit PVAT-derived chemerin-induced vasoconstriction in isolated rat arteries [13, 47]. These findings are consistent with studies demonstrating that ex vivo incubation with CCX832 attenuates vascular contraction in isolated rat mesenteric arteries and thoracic aortas, as well as in human pulmonary and coronary arteries [176, 177], highlighting its broad vasodilatory potential via CMKLR1 antagonism (Fig. 2).

## Conclusion

PVAT plays a critical role in the regulation of vascular tone and function through its paracrine and endocrine properties and is increasingly recognized as the “fourth layer” of the blood vessel wall. This role becomes particularly significant in the context of obesity-related cardiovascular complications, where PVAT exerts distinct influences on vascular homeostasis.

While circulating chemerin is produced primarily by the liver, accumulating evidence suggests that PVAT-derived chemerin plays a pivotal role in the pathogenesis of hypertension and other obesity-associated vascular diseases. Given its local effects on vascular function, elucidating the specific role of chemerin produced by PVAT is essential for the development of targeted pharmacological interventions for metabolic and cardiovascular disorders.

To achieve this goal, there is an urgent need to develop PVAT-specific genetic mouse models, including chemerin or CMKLR1 knockout and overexpression systems, to elucidate the precise signaling mechanisms involved. Using such models, future research should aim to address the following critical questions:

- What is the exact role of PVAT-derived chemerin in regulating both PVAT function and vascular wall biology?
- What are the specific isoform profiles of chemerin in PVAT and adjacent vascular tissue?
- Why do chemerin molecules derived from the liver and PVAT exhibit differential vascular effects?

Despite the current gaps in knowledge, existing studies have significantly advanced our understanding of the vascular actions of chemerin, particularly the contribution of chemerin to the pathophysiology of obesity-related vascular dysfunction. Moreover, several promising chemerin-targeted therapeutic strategies—including receptor antagonists, isoform-specific modulators, and antisense oligonucleotides—have been proposed.

Future research efforts should focus on the selective modulation of PVAT-derived chemerin, which holds strong potential for the development of novel therapies to combat obesity-associated vascular diseases.

## Data Availability

No datasets were generated or analysed during the current study.
